# Effect of an eHealth Intervention to Reduce Sickness Absence Frequency Among Employees With Frequent Sickness Absence: Randomized Controlled Trial

**DOI:** 10.2196/10821

**Published:** 2018-10-23

**Authors:** Annette Notenbomer, Corné Roelen, Johan Groothoff, Willem van Rhenen, Ute Bültmann

**Affiliations:** 1 Division Community and Occupational Medicine, Department of Health Sciences University Medical Center Groningen University of Groningen Groningen Netherlands; 2 Arbo Unie Utrecht Netherlands; 3 Business Universiteit Nyenrode Breukelen Netherlands

**Keywords:** occupational health, sick leave, randomized controlled trial, adult, occupational health physicians, eHealth

## Abstract

**Background:**

Frequent sickness absence—that is, 3 or more episodes of sickness absence in 1 year—is a problem for employers and employees. Many employees who have had frequent sickness absence in a prior year also have frequent sickness absence in subsequent years: 39% in the first follow-up year and 61% within 4 years. Moreover, 19% have long-term sickness absence (≥6 weeks) in the first follow-up year and 50% within 4 years. We developed an electronic health (eHealth) intervention, consisting of fully automated feedback and advice, to use either as a stand-alone tool (eHealth intervention–only) or combined with consultation with an occupational physician (eHealth intervention–occupational physician).

**Objective:**

This study aimed to evaluate the effect of the eHealth intervention, with or without additional occupational physician consultation, to reduce sickness absence frequency for employees with frequent sickness absence, versus care as usual (CAU).

**Methods:**

This study was a three-armed randomized controlled trial. Employees with frequent sickness absence received invitational letters, which were distributed by their employers. The primary outcome measure was the number of register-based sickness absence episodes 12 months after completing the baseline questionnaire. Secondary outcome measures were register-based total sickness absence days and self-assessed burnout, engagement, and work ability. In a process evaluation 3 months after baseline, we examined adherence to the intervention and additional actions such as general practitioner and occupational physician visit, communication with the manager, and lifestyle change.

**Results:**

A total of 82 participants were included in the analyses, 21 in the eHealth intervention–only group, 31 in the eHealth intervention–occupational physician group, and 30 in the CAU group. We found no significant difference in sickness absence frequency between the groups at 1-year follow-up. Sickness absence frequency decreased in the eHealth intervention–only group from 3 (interquartile range, IQR 3-4) to 1 episode (IQR 0.3-2.8), in the eHealth intervention–occupational physician group from 4 (IQR 3-5) to 3 episodes (IQR 1-4), and in the CAU group from 3 (IQR 3-4) to 2 episodes (IQR 1-3). For secondary outcomes, we found no significant differences between the intervention groups and the control group. The process evaluation showed that only 3 participants from the eHealth intervention–occupational physician group visited the occupational physician on invitation.

**Conclusions:**

Among employees with frequent sickness absence, we found no effect from the eHealth intervention as a stand-alone tool in reducing sickness absence frequency, nor on total sickness absence days, burnout, engagement, or work ability. This might be due to low adherence to the intervention because of insufficient urgency to act. We cannot draw any conclusion on the effect of the eHealth intervention tool combined with an occupational physician consultation (eHealth intervention–occupational physician), due to very low adherence to the occupational physician consultation. An occupational physician consultation could increase a sense of urgency and lead to more focus and appropriate support. As this was the first effectiveness study among employees with frequent sickness absence, strategies to improve recruitment and adherence in occupational eHealth are included.

**Trial Registration:**

Netherlands Trial Register NTR4316; http://www.trialregister.nl/trialreg/admin/rctview.asp?TC=4316 (Archived by WebCite at http://www.webcitation.org/713DHhOFU).

## Introduction

### Background

Frequent sickness absence (SA)—that is, 3 or more episodes of SA in a year,—is common in the Dutch working population and poses a problem for both employers and employees [1]. The prevalence of frequent SA was 5.8% in 2015 and 6.1% in 2016 among more than 600,000 employees working in small- to medium-sized companies under contract with a Dutch national occupational health service (OHS). In the Netherlands, the costs related to frequent SA can be estimated to be at least 100 million euros per year (US $123 million) [2]. Frequent SA is often a precursor of future frequent SA; Koopmans et al found that 39% of employees with frequent SA had frequent SA again in the following year and 61% within 4 years [3]. In addition, there is a risk of high future costs due to long-term SA, as 19% of frequent absentees have long-term SA (≥6 weeks) in the first follow-up year and 50% in the 4 years following a year with frequent SA [3]. Employers have to redirect work tasks to other employees and are faced with work planning problems. Employees with frequent SA can suffer from increasing tensions with colleagues [4] who are burdened with an increased workload and overwork. Furthermore, frequent absentees are at increased risk of being dismissed [5]. In a literature review, Beemsterboer et al [6] found that poorer health, poorer working conditions, greater physical and mental workload, female gender, greater alcohol consumption, and smoking were related to a higher SA frequency. In contrast, better job resources, better working relations, greater motivation (work pleasure), older age, being married, having a satisfactory private life, and higher education were associated with a lower SA frequency. Other studies found that poor health, chronic diseases, and reduced work ability were related to frequent SA [7-10]. On the basis of a focus group study among frequent absentees, we reported earlier that high job demands and low job resources, particularly low support from management, were related to frequent SA [11]. Frequent absentees also mentioned home demands, poor health, chronic illness, and unhealthy lifestyles to be related to frequent SA [11]. The results of these studies suggest that frequent SA is a multifaceted phenomenon.

To reduce SA frequency among employees with frequent SA, an intervention is needed to address this wide range of issues. To reduce frequent SA and feel better, employees with frequent SA prefer to seek adequate (medical) help themselves [11]. They want help only when self-management fails [11]. Among published interventions to reduce SA, several studies showed successful reduction of SA in employees at high risk of future SA through face-to-face structured consultations with occupational health professionals [12-15]. However, consultations can be time-consuming and costly. In contrast, electronic health (eHealth) interventions are low in cost and appeal to self-management. The number of studies on eHealth interventions (EHIs) has grown rapidly in the last few years. Studies among patient or general populations have shown that EHIs enhance health and well-being, improve lifestyle, and increase self-management for a broad range of diseases or risk factors [16-21]. In occupational health, however, studies on EHIs are relatively scarce. eHealth studies among employees focus mainly on lifestyle change, stress reduction, or mental health improvement. Studies on effectiveness of EHIs on absenteeism measures are few. In a randomized controlled trial (RCT) among employees at high risk of SA due to depression, Beiwinkel et al tested a 12-week eHealth program and compared the results with those of a waiting-list control group that received written psychoeducation. Although both groups showed a high reduction in SA frequency (intervention group: 67% and control group: 83%), there was no statistical difference in SA frequency between the groups [22]. Ebert et al [23] found a reduction of depressive symptoms among a group of teachers through an internet-based problem-solving training but no difference between the intervention and control groups on the secondary outcome measure self-rated absenteeism. Other studies showed that a self-guided internet-based stress management intervention resulted in a significant reduction of perceived stress in a group with increased stress but no reduction in absenteeism [24-26]. Volker et al [27] reported an earlier return to work in the intervention group among sick-listed employees with common mental disorders (hazard ratio 1.39, 95% CI 1.03-1.87). During a 1-year follow-up period, the intervention group received a blended EHI, compared with the care as usual (CAU) group. The blended EHI consisted of a mix of personalized e-modules in combination with guidance by a specially trained occupational physician (OP). All these EHIs have the use of questionnaires in common for a targeted intervention and elements of self-management.

To our knowledge, no EHIs have as yet been designed to reduce SA frequency among frequent absentees. We developed an EHI tool to advise employees with frequent SA as to how they could improve health and self-management. The theoretical framework for this EHI tool was based on the Job Demands-Resources (JD-R) model [28]. The JD-R model relates psychosocial work characteristics to outcome measures such as burnout, engagement, productivity, and SA [29-32] and provides keys for guidance and support [11,32]. We used tools from the mental health guidelines of the Netherlands Society for Occupational Physicians (NVAB) [33]. Furthermore, the EHI tool was based on the determinants of frequent SA according to focus group participants and their suggestions on how to reduce frequent SA, such as communication with management [11]. The intervention addressed these elements item by item.

In the Netherlands, OPs often play a role in work-related interventions to reduce SA as they are experts in health, work, SA, and prevention. They can also address factors from the JD-R model, advising (temporary) accommodations to reduce job demands or increase job resources. The role of OPs in effective reduction of SA [12,14,15] led us to include a blended-care study arm, combining the EHI tool with a consultation with the OP.

### Objective

The main objective of this study was to evaluate the effectiveness of the EHI tool without (EHI-only) and with OP consultation (EHI-OP), compared with CAU, on SA frequency among employees with frequent SA. Secondary outcomes were the total number of SA days, burnout, work engagement, and work ability at 1-year follow-up. We conducted a process evaluation to evaluate adherence to the intervention.

## Methods

### Design and Participants

This study was designed as a three-armed RCT and registered in the Dutch trial register (NTR 4136). The Medical Ethics Committee (METc) of the University Medical Center Groningen approved the study (METc 2013/131). Employees participated voluntarily and signed an informed consent form (see Multimedia Appendix 1). The report is based on the CONSORT eHealth checklist [34] (see Multimedia Appendix 2).

Inclusion criteria were employees with frequent SA, that is, 3 or more episodes of SA in the year before recruitment, irrespective of the causes or duration of sick leave. Exclusion criterion was inability to complete a questionnaire in Dutch.

Study participants were recruited from 21 Dutch organizations staffing more than 100 employees between December 2013 and December 2014. Of the participating organizations, 7 were in industry, 5 in commercial services, and 9 in public services. The first author (AN) had prepared a list of all frequent absentees in the participating organizations (source population), based on the occupational health service register. All employees with frequent SA received from us invitational letters combined with informed consent forms, which were distributed by their employers. The letters contained logos from both the University of Groningen and the OHS. Upon signing the informed consent form, we sent a personal URL code, which provided access to the Web-based questionnaire at baseline (T0). The questionnaire included validated scales measuring secondary outcome measures such as burnout, engagement, and work ability as well as potential determinants of frequent SA as found in the focus group study: job demands, job resources, health, chronic diseases, lifestyle aspects, and a few open questions on health and frequent SA.

Upon completion of the baseline questionnaire, the intervention groups received fully automated personal advice. The control group was thanked for participation upon completion of the questionnaire. All participants were invited to fill out a process evaluation 3 months after T0 and a short questionnaire 1 year after baseline (T1), with questions on the outcome measures.

### Intervention

#### Intervention Group 1: eHealth Intervention Only (EHI-only)

The main scope of the intervention was self-management with help from relevant others, for example, the employer, OP, and general practitioner (GP). The intervention consisted of immediate fully automated personalized Web-based feedback, item by item. The addressed items were job demands (work pace, emotional demands, and work-home interference), job resources (feedback, learning opportunities, supervisor support, coworker support, and autonomy), burnout, engagement, work ability, general health, chronic diseases, psychological health, lifestyle, and body mass index. For an overview of the intervention elements per item, see Multimedia Appendix 3. The feedback per item consisted of the score, interpretation of the score, general advice on possibilities to tackle this issue (in case of a poor score), reference to relevant internet sites for more information, further diagnostic tests or treatment, and referral to people who could help (depending on the issue: manager, colleagues, human resource management [HRM], OP, social worker, or GP). The advice often contained a link to documents with more detailed advice. The advice was based on NVAB guidelines, occupational health care practice, and suggestions from focus group participants with frequent SA from a prior study [11]. Cut-off points were either the existing cut-off points of the scales or the seventy-fifth percentile of a large reference group who participated in OHS health surveillance checks. Participants who scored well on a particular scale received feedback that they had scored well and no specific actions were needed.

#### Intervention Group 2: eHealth Intervention + Occupational Physician Consultation (EHI-OP)

Participants in intervention group 2 received the same advice and documents as the EHI-only group but were invited by email to a preventive advisory consultation with the OP. The email contained the name of the OP and the telephone number of the OP’s secretary to make an appointment.

The OPs from the 21 participating organizations received written information on the study (see Multimedia Appendix 4) and a personal explanation by the first author (AN) about the goal of the study and the possibility of consultations with participants. Moreover, AN explained that what was expected in this preventive consultation was the same as in preventive consultations initiated by the employee in nonresearch situations: mainly participants’ questions on health and SA in relation to work and how to influence the employee’s health or (work) situation. This could lead to making a joint plan-of-action, but it was not obligatory. Standard time for this preventive consultation was 30 min.

#### Control Group: Care as Usual

The control group received neither personalized advice nor support from the OP or researchers upon completion of the Web-based questionnaire. CAU consisted of consultation with the OP at the request of the employer or control group participant. In case of long-term SA, participants were invited for a consultation with the OP to certify SA within 6 weeks of reporting sick [35].

#### Pilot Test of eHealth Intervention

We pilot tested the EHI tool in 12 frequent absentees from 3 nonparticipating organizations. We used their feedback on technical issues and understandability to improve the EHI tool. After finalization of the tool, we made no changes in the contents.

### Primary Outcome Variable

The primary outcome measure was the number of register-based SA episodes 12 months after completing the Web-based baseline questionnaire. At 1-year follow-up, the incident number of SA episodes was retrieved at the individual level from the occupational health service register, in which SA was recorded from the first day of sick leave to the day of return to work.

### Secondary Outcome Variables

The number of days of all SA episodes was cumulated to a total number of SA days at 1-year follow-up. Burnout, work engagement, and work ability were measured at 1-year follow-up.

Burnout was measured with the 9-item Utrecht Burnout Scale (UBOS) measuring emotional exhaustion and cynicism on a 7-point frequency scale ranging between never (=0) and always (=6) [36]. A total UBOS-9 score (Cronbach alpha=.92) was calculated by summing the item scores, with higher scores representing higher levels of burnout. The scale included the dimensions emotional exhaustion (5 items) and cynicism (4 items).

Work engagement was measured with the 9-item Utrecht Work Engagement Scale (UWES) [37]. The UWES scale’s vigor (3 items), dedication (3 items), and absorption (3 items) were scored on a 7-point frequency scale ranging from “never” (=0) to “always” (=6). A total UWES-9 score was calculated by summing the item scores (Cronbach alpha=. 95), with higher scores representing more work engagement.

Work ability was investigated with the first item of the Work Ability Index (WAI), asking for current work ability compared with lifetime best on a 0 to 10 scale. This single-question work ability score has been reported to be a reasonable alternative to the complete WAI for measuring work ability in working populations [38,39].

### Work and Population Characteristics

We assessed the following items to provide targeted intervention advice. Job demands (work pace, emotional demands, and work-home interference) and job resources (feedback, learning opportunities, supervisor support, coworker support, and autonomy) were measured with the short scales of the Questionnaire on the Experience and Evaluation of Work [40]. General health was measured with the Short-Form Health Survey, the single-item question: “In general, would you say your health is excellent (=5), very good (=4), good (=3), fair (=2), or poor (=1)?” This item has been associated with physicians’ assessments of health, morbidity measures, and utilization of health services [41] and is comparable with longer instruments [42]. The presence of chronic diseases was assessed with the item “Do you have a chronic disease that already lasts >3 months?” (yes/no). The Alcohol Use Disorder Identification Test [43] was used to measure alcohol consumption. Physical exercise was measured with Dutch Norm Healthy Moving [44] and Fitnorm [45]. Smoking was assessed by a single-item question on smoking (yes/no) and relaxation with 2 single-item questions: “Do you have at least half an hour relaxation every day?” and “Do you regularly take a break at work?” Response options were “hardly every/never,” “regularly,” and “(almost) always.” Additionally included were the questions “Do you find it a problem that you are frequently on sick-leave?” (yes, no, do not know), “Does your supervisor find it a problem that you are frequently on sick-leave?” (yes, no, do not know), and “Do your colleagues find it a problem that you are frequently on sick-leave?” (yes, no, do not know, some do/some do not). An open question at the end of the questionnaire requested other relevant information (“What else plays a role in your health and SA that has not yet been covered?”).

### Process Evaluation

In a process evaluation 3 months after baseline, adherence to the intervention was measured in the EHI groups, assessing reading the advice provided by the eHealth tool and undertaking actions. Actions assessed were a consultation with the OP, GP, specialists, paramedics, or psychologist and additional actions such as tackling sources of stress, tackling work-related problems, and having a conversation at work about work-related problems or solutions, lifestyle changes, and other actions (open question). Participants from the control group also received a process evaluation, with questions on OP consultation and additional actions, for example, visit to GP, physiotherapist, psychologist, or other paramedics; lifestyle changes; or consultations with management, HRM, or occupational health providers to seek solutions for work-related problems.

### Sample Size

In a pilot study, we found that frequent absentees had on average 3.79 (SD 1.27) SA episodes in 2013 to 2014 in the total employee population of a large Dutch OHS. RCT intervention studies that include SA frequency as an outcome measure are scarce. No scientifically based intervention effect was available as this was the first intervention study among employees with frequent SA on SA frequency. The RCT studies from Duijts et al [13] and Kant et al [15] are the closest scientific approaches to this intervention study, although targeted at a different population. Applying their results to our study on frequent absentees, we aimed in the original trial protocol for a reduction of 0.5 episodes (Cohen *d*=0.39) with our focused intervention. On the basis of an alpha of .05 (two-tailed) and a power of 80%, a sample size of 103 was needed [46]. After further consideration, we included in the submission to the METc, before the start of the study, a second sample size calculation to detect a difference of 1 SA episode per year (Cohen *d*=0.79). This was based on our practice-based knowledge of relevant intervention effects in an occupational health setting. This calculation showed that we needed a minimum of 27 per group [46].

### Randomization

The source population (N=825) was prerandomized into 3 arms: intervention group 1 (EHI-only; n=270), intervention group 2 (EHI-OP; n=279), and control group (n=276) by random integers [47]. We randomized the entire source population as the software provider was only able to generate and provide URLs groupwise.

### Blinding

Participants were allocated to the intervention groups and control group before the study started. They were blinded for the group to which they were allocated until completion of the Web-based questionnaire, whereupon they did (intervention groups) or did not (control group) receive a personalized advice. The first author (AN) knew to which group each individual belonged. SA data were retrieved and analyzed by another author (CR) who did not know to which group each individual belonged.

### Statistical Analyses

All statistical analyses were conducted in IBM SPSS Statistics for Windows, version 24 (released 2016; IBM Corp. Armonk, NY). Baseline data on primary and secondary outcomes were register-based or provided by participants. Missing information from follow-up assessments was imputed using baseline observation carried forward.

#### Analysis of 3 Study Arms

First, we investigated differences in outcomes at T1 between the EHI-only group, the EHI-OP group, and the CAU group according to the intention-to-treat principle. Due to the non-normal distribution of incident SA episodes and days, we investigated differences by using the nonparametric Kruskal-Wallis test.

#### Analysis of Combined Intervention Groups Versus Control Group

The intervention groups (EHI-only and EHI-OP) were merged, as all participants from these study arms had access to the same EHI and only 3 (13%) participants from the EHI-OP group additionally consulted the OP upon invitation. We investigated the differences between the outcomes of the combined intervention groups and the control group by using the nonparametric Mann-Whitney U test.

## Results

### Descriptive Statistics

**Figure 1:**
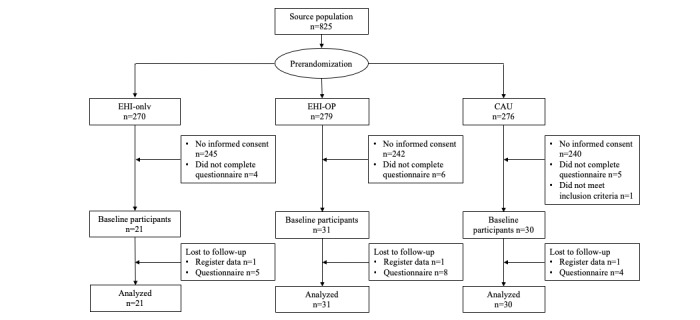
Flowchart of participants. CAU: care as usual; EHI-only: study arm with only eHealth intervention; EHI-OP: study arm with eHealth intervention and invitation for OP consultation.

All 825 eligible employees with frequent SA from 21 participating organizations (the source population) were randomized. A total of 2 reorganizing organizations withdrew from the study after receiving negative reactions from their employees (n=122 eligible employees). In 6 other organizations, none of the eligible employees (n=163) decided to participate. From the other 13 organizations, 525 employees did not send an informed consent form. Some employees (n=15) filled out the consent form but failed to complete the baseline questionnaire. One employee was misregistered as having frequent SA. After exclusion of these 743 eligible employees, the study included 82 participants at baseline. SA-registered data of 3 participants were lost to follow-up due to temporary contracts that ended during the follow-up period. In total, 17 participants did not fill out the last questionnaire. Finally, 21 participants were included in the EHI-only group, 31 in the EHI-OP group, and 30 in the CAU group in the analysis. Figure 1 provides an overview of the recruitment flow.

**Table 1:** Characteristics of participants.

Participant characteristics			EHI-only^a^ (N=21)	EHI-OP^b^ (N=31)	CAU^c^ (N=30)
**Sociodemographic characteristics**					
	Age (years), mean (SD)		44.9 (10.1)	45.9 (11.4)	46.9 (10.9)
	Gender (male), n (%)		7 (33)	10 (32)	10 (33)
	Marital status (married or living together), n (%)		18 (86)	23 (74)	21 (70)
	**Educational level, n (%)**				
		Low	4 (19)	3 (10)	2 (7)
		Intermediate	9 (43)	10 (32)	11 37)
		High	8 (38)	18 (58)	17 (57)
**Work-related characteristics**					
	Irregular work (eg, night shift), n (%)		5 (24)	6 (19)	5 (17)
	Years with current employer, mean (SD)		13. (8.9)	14.4 (10.9)	15.1 (11.6)
	Hours per week, mean (SD)		31.1 (7.4)	34.9 (8.7)	33.1 (11.1)
	Workload, mean (SD)		2.5 (0.6)	2.7 (0.7)	2.5 (0.9)
	Emotional demands, mean (SD)		2.2 (1.0)	1.9 (0.8)	1.9 (0.6)
	Conflict at work (current), n (%)		3 (14)	1 (3)	3 (10)
	Work-home interference, mean (SD)		1.5 (0.4)	1.8 (0.8)	1.7 (0.8)
	Performance feedback, mean (SD)		3.1 (1.1)	3.1 (1.0)	3.1 (1.0)
	Opportunities to learn and develop, mean (SD)		2.7 (1.0)	2.6 (1.0)	2.6 (1.1)
	Support manager, mean (SD)		2.9 (1.1)	2.8 (1.2)	3.1 (1.1)
	Support colleagues, mean (SD)		3.5 (0.8)	3.7 (1.0)	3.4 (1.2)
	Autonomy, mean (SD)		3.3 (1.0)	3.7 (0.9)	3.1 (1.0)
**Health related, n (%)**					
	**Health perception (short form health survey 1-item [SF-1])**				
		Bad	0 (0)	3 (10)	1 (3)
		Fair	4 (19)	7 (23)	14 (47)
		Good	14 (67)	14 (45)	11 (37)
		Very good	2 (10)	5 (16)	3 (10)
		Excellent	1 (5)	2 (7)	1 (3)
	Chronic disease		7 (35)	12 (40)	8 (28)

^a^EHI-only: study arm with only eHealth intervention.

^b^EHI-OP: study arm with eHealth intervention and invitation for occupational physician consultation.

^c^CAU: care as usual.

Table 1 shows the characteristics of the 82 analyzed participants. The mean age was 46 years. The percentage of men was 33% (27/82)and 76% (62/82) were married or living together. Few participants had low education: 19% (4/21) of the EHI-only group, 10% (3/31) of the EHI-OP group, and 7% (2/30) of the CAU group. Many participants found frequent SA a problem for themselves (58/82, 71%), but a very low percentage reported that they thought that this was the case for their managers (15/82, 18%) or colleagues (11/82, 13%). The information on self-reported factors playing a role in health and frequent SA could be categorized into 5 main categories: type of disease (eg, chronic disease and migraine, 28/82, 34%), high job demands (12/82, 15%), low job resources (4/82, 5%), home demands (7/82, 9%), and imbalance between demands and capacity (4/82, 5%). An additional 26 participants (32%) did not answer this open question.

### Analysis of 3 Study Arms

#### Primary Outcome

There was no significant difference in reduction of SA frequency between the 3 study arms (Kruskal-Wallis: *P*=.66). All 3 groups, EHI-only, EHI-OP, and CAU, showed a significant reduction in SA frequency over time (*P* values of respective Wilcoxon signed rank tests: EHI-only: *P*=.006, EHI-OP: *P<*.001, and control group: *P*<.001). Where all participants had frequent SA at baseline, at 1-year follow-up, 5 participants in the EHI-only group (5/21, 25%) had frequent SA, 16 participants in the EHI-OP group (16/31, 52%), and 12 in the CAU group (12/30, 40%, data not shown). Table 2 shows that there was no significant difference in SA frequency between the EHI groups and the CAU group at 1-year follow-up.

#### Secondary Outcomes

All 3 groups showed a reduction in total SA days over time. No significant difference was found between the EHI and CAU groups in the total number of SA days at 1-year follow-up (Table 2). In the EHI-only group, 3 (15%) had long-term SA (ie, ≥42 consecutive days) during 1-year follow-up, 7 in the EHI-OP group (23%), and 8 in the CAU group (28%, data not shown). We found no significant differences between the EHI groups and CAU group in burnout and engagement. Work ability was significantly lower in the EHI-OP group at 1-year follow-up compared with the other groups.

### Analysis of Combined Intervention Groups Versus Control Group

#### Primary Outcome

The combined intervention groups (EHI groups) and the CAU group showed a reduction in SA frequency over time (Table 3). At 1-year follow-up, 21 participants (42%) in the combined EHI groups again had frequent SA as well as 12 in the CAU group (41%, data not shown). Table 3 shows that there was no significant difference in SA frequency between the EHI groups and the CAU group at 1-year follow-up.

#### Secondary Outcomes

The combined intervention groups and the control group showed a reduction in total SA days over time. There was no significant difference between the EHI groups and the CAU group in the total number of SA days at 1-year follow-up (Table 3). In the EHI groups, 10 participants (20%) had long-term SA (ie, ≥42 consecutive days) during 1-year follow-up, compared with 8 in the CAU group (40%, data not shown). No significant differences were found between the EHI groups and CAU group in burnout, engagement, or work ability.

**Table 2:** Results of 3 study arms.

Outcome at 1-year follow-up		Median T0 (IQR^a^ 25-75%)	Median T1 (IQR 25-75%)	*P* value (Kruskal-Wallis)^b^
**Frequency of sickness absence episodes**				.43
	EHI-only^c^	3 (3-4)	1 (0.5-3.5)	
	EHI-OP^d^	4 (3-5)	3 (1-4)	
	CAU^e^	3 (3-4)	2 (1-3)	
**Total number of sickness absence days**				.15
	EHI-only	22 (14.5-37.5)	5 (1-25)	
	EHI-OP	17 (8-34)	11 (4-36)	
	CAU	20.5 (11.5-38.8)	12.5 (7.0-73.5)	
**Burnout (range 0-6)**				.30
	EHI-only	0.9 (0.3-1.5)	1.2 (0.2-1.6)	
	EHI-OP	1.4 (0.6-2.0)	1.3 (0.5-2.3)	
	CAU	1.3 (0.5-2.2)	1.4 (0.8-2.2)	
**Engagement (range 0-6)**				.27
	EHI-only	3.6 (2.4-4.5)	3.9 (2.8-4.8)	
	EHI-OP	3.7 (2.6-4.7)	3.4 (2.6-4.1)	
	CAU	2.8 (2.1-4.3)	3.3 (2.4-3.9)	
**Work ability (range 0-10)**				.01
	EHI-only	8 (7-9)	8 (8-9)	
	EHI-OP	8 (8-9)	7 (7-8)	
	CAU	8 (7-9)	7.5 (7-8)	

^a^IQR: interquartile range.

^b^*P* values for differences between the 3 study arms at T1.

^c^EHI-only: study arm with only eHealth intervention.

^d^EHI-OP: study arm with eHealth intervention and invitation for occupational physician consultation.

^e^CAU: care as usual.

**Table 3:** Results of analysis with combined intervention groups versus control group.

Outcome at 1-year follow-up		Median T0 (IQR^a^ 25-75%)	Median T1 (IQR 25-75%)	*P* value (Mann-Whitney U)^b^
**Frequency of sickness absence episodes**				.91
	EHI groups^c^	3.5 (3-4.8)	2.4 (1-4)	
	CAU^d^	3 (3-4)	2 (1-3)	
**Total number of sickness absence days**				.19
	EHI groups	19 (10.3-37)	8.7 (2.3-31.5)	
	CAU	20.5 (11.5-38.8)	12.5 (7-73.5)	
**Burnout (range 0-6)**				.29
	EHI groups	1.2 (0.5-1.9)	1.3 (0.4-2.1)	
	CAU	1.3 (0.5-2.2)	1.4 (0.8-2.2)	
**Engagement (range 0-6)**				.16
	EHI groups	3.6 (2.6-4.6)	3.6 (2.6 -4.4)	
	CAU	2.8 (2.1-4.3)	3.3 (2.4-3.9)	
**Work ability (range 0-10)**				.23
	EHI groups	8 (7-9)	7.4 (7-9)	
	CAU	8 (7-9)	7.5 (7-8)	

^a^IQR: interquartile range.

^b^*P* values for the combined eHealth intervention groups versus the care as usual group at T1.

^c^EHI groups: combined eHealth intervention groups.

^d^CAU: care as usual.

### Process Evaluation: Adherence to the Intervention and Additional Actions

In a process evaluation 3 months after baseline, all participants received a questionnaire focusing on adherence to the intervention and additional actions they had taken. In total, 55 (70%) participants responded to the process evaluation.

A total of 3 participants out of 30 (10%) in the EHI-OP group reported that they had consulted the OP upon study invitation. Moreover, 2 participants from this study arm had seen the OP at a later moment (at the initiative of their employer) because of longer SA. A total of 2 participants from the EHI-only group (10%) and 3 from the control group (10%) reported having consulted the OP. All participants who had visited the OP were satisfied with the consultation.

In the EHI-only group, 54% (7/13) process evaluation responders reported that they had received the digital advice and 72% (13/18) in the EHI-OP group. Of the 7 receivers in the EHI-only group, 4 (57%) had read the additional documents provided. This was the case for 46% (6/13) receivers in the EHI-OP group. No participant made a plan-of-action as provided in the digital tool. For a schematic overview of adherence to the intervention and additional actions, see Table 4.

Participants from all groups—intervention and control—took additional actions. A total of 16 participants (29%) took action at work (4/13 in the EHI-only group, 31%; 5/18 in the EHI-OP group, 28%; and 7/24 in the control group, 29%). Overall, we observed no marked differences between actions in the EHI groups and the CAU group. A total of 4 participants from the EHI-only group (31%) and 9 from the EHI-OP group (50%) had taken no (new) actions since study participation. Many participants reported why they had not undertaken any further actions, including OP consultations, for example, having already taken a lot of actions before the study, still undertaking actions they had started before the study, not acknowledging the added value of the intervention when knowing the problem is not work-related, being too busy, low urgency, and optimism about their future health and SA.

**Table 4:** Overview of actions per group according to process evaluation.

Actions undertaken		EHI-only^a^ (n=13)	EHI-OP^b^ (n=18)	CAU^c^ (n=24)
Participation rate in process evaluation, 3 months after intervention, %		65	60	83
Digital scores and advice received, n		7	13	N/A^d^
**Digital advice, n**				N/A
	Fully read	7	9	
	Partially read	N/A	3	
	Not read	N/A	1	
Digital documents read, n		4	6	N/A
Visits to occupational physician according to process evaluation, n		2	5	3
Visit to general practitioner or medical specialist, n		1	4	3
Visit to psychologist, n		1	1	1
Tackle sources of stress, n		2	0	2
Tackle or discuss problems and solutions at work, n		4	5	7
Change lifestyle, n		3	5	7

^a^EHI-only: study arm with only eHealth intervention.

^b^EHI-OP: study arm with eHealth intervention and invitation for OP consultation.

^c^CAU: care as usual.

^d^N/A: not applicable.

## Discussion

### Principal Findings

The primary aim of this study was to evaluate the effectiveness of the EHI tool without OP consultation (EHI-only) or with OP consultation (EHI-OP) on SA frequency among employees with frequent SA compared with CAU. The secondary aim was to evaluate the effectiveness of the interventions (EHI groups) on the total number of SA days, burnout, engagement, and work ability. There was no significant difference in SA frequency during follow-up between the EHI groups and the CAU group. SA frequency was lower at T1 compared with T0 for all groups. We also found no significant difference on total SA, burnout, and engagement at T1 between the EHI groups and CAU group. Work ability was lower in the EHI-OP study arm than in the CAU group at 1-year follow-up in the “analysis of 3 study arms, but not in the analysis comparing the combined intervention groups versus the control group.” The combination of the EHI tool with an OP consultation was not tested, as only 3 people consulted the OP upon invitation. The results from the EHI-OP group represent more the effect of the EHI intervention as a stand-alone tool than the intended blended care.

Our findings on SA frequency are in line with previous results of RCTs on the effect of non-EHIs. Duijts et al [13] reported that a coaching intervention (7 to 9 sessions of 1 hour) did not result in a significant difference of SA episodes in the intervention group compared with the control group. However, the number of SA days at follow-up was significantly lower. Likewise, Kant et al [15] found no significant differences in SA episodes between intervention and control groups in an intervention with structured early consultations with the OP. They found a significantly lower SA duration in the follow-up period in a per-protocol analysis, but not in the intention-to-treat analyses. Although the intervention groups in our study also showed lower total SA days at 1-year follow-up compared with the control group, we found no significant difference between the groups.

That no significant differences were found between groups might be explained by too few participants in the intervention and control groups. Another explanation for finding no effect is that we may have included participants with more serious conditions than in the general population of frequent absentees, that is, conditions that are more difficult or impossible to solve or treat. One indication of having a selection of frequent absentees with more severe conditions is that some participants joined the study after having started a period of long-term SA. Another indication of selection of frequent absentees with more severe conditions is the incidence of long-term SA. In our study, 28% (8/30) in the control group had long-term SA (≥42 days), as opposed to 19% in an earlier longitudinal study on the risk of long-term SA in 4126 frequent absentees [2]. Possibly, the use of the logo of an OHS may have contributed to a selection bias toward participants with more severe conditions as OHS in the Netherlands mostly focus on employees with long-term SA.

The EHI-OP intervention might have been more effective if more frequent absentees had visited the OP for preventive advisory consultations. The resulting increased awareness of the high-risk of future long-term SA could have motivated absentees to undertake more actions to improve their situation. The OP could also have helped them to prioritize these actions by working with them to develop a structured plan of action. Moreover, the OP could have referred them to other professionals, such as a company social worker or psychologist. According to a recent meta-analysis by Heber et al [48], guided computer-based interventions, combining computer based-intervention with human written guidance, may be more effective than stand-alone EHIs. In meta-analyses, Hutchesson et al found effectiveness of EHIs to be enhanced by the addition of face-to-face group or individual sessions or extra technologies such as self-monitoring tools, email counseling, or online group discussions [49]. Reasons for frequent absentees not to accept the invitation to consult with an OP included mistrust, insufficient urgency to take (additional) action, not acknowledging the added value of the OP when knowing the (private) problem, being too busy, and optimism about their future health and SA.

Another explanation for our results may be low adherence to the EHI tool itself; in the process evaluation, 15 participants (15/55, 27%) reported not having received or (fully) read the digital advice. Only a few participants read the additional documents. Many (23/55, 42%) did not take additional actions such as seeking advice from general practitioners, paramedics, or discussions with the employer about work problems. Although 71% (58/82) of the participants considered their frequent SA to be a problem, only 18% (15/82) of participants considered frequent SA to be a problem for the manager and only 13% (11/82) for (some of) the colleagues. Overall, it seems that frequent absentees did not feel a sense of urgency, either intrinsically or in response to external pressure.

Another reason for no effect may be that the control group was made aware of frequent SA and the risk of long-term SA by the invitational study leaflet and the Web-based questionnaire. The increased awareness may have stimulated the control group to take actions aimed at reducing their SA frequency; the process evaluation shows that they indeed did take actions.

### Strengths and Limitations

This is the first RCT to study the effect of an EHI tool on employees with frequent SA. The baseline questionnaire used instruments and scales validated for use in occupational health care. As the SA measures were register-based, there was limited attrition. This study was undertaken in the Netherlands, limiting cross-country generalizability. The advantage is that all participants fall under the same regulations, with registration from the first SA day and salary payment also from the first SA day, regardless of the cause of SA.

As studies that include SA frequency as outcome measure are scarce and do not target the group of frequent absentees, we had no information on a realistic intervention effect. We included 2 sample size calculations in the study protocol as registered with the METc, 1 based on intervention studies in a more heterogeneous population and 1 based on our practice-based knowledge on a relevant intervention effect in an occupational health setting. Although aiming for the highest sample size, due to low participation, we accepted the lower sample size of 27 participants per group. In hindsight, this sample size was low and may be the reason for not finding effect.

Only 9.9% (82/825) eligible frequent absentees participated in the study. The absentees are probably not aware that frequent SA often recurs and may pose a risk of future long-term SA [11], regardless of the information in the invitational leaflet. We dealt with potential selection bias by using prestudy randomization: participants were allocated to intervention and control groups before the study started; they were blinded for the group to which they were allocated until they did or did not receive the digital advice. The low participation rate may have affected the generalizability of the results.

Few participants in the EHI-OP group visited the OP for advice and guidance. Thereupon, we conducted an analysis of the “combined intervention groups versus control group,” which led to higher statistical power for testing the EHI than in the “analysis of the 3 study arms.” However, we could not draw conclusions on the effectiveness of blended care (EHI-OP), as too few participants consulted the OP. The reason for low adherence to the blended intervention (EHI-OP) may be the voluntary character of the invitation, without any support or stimulation from researchers, employer, or OHS to visit the OP, in combination with an insufficient sense of urgency of the frequent absentee.

### Learnings and Implications for Future Research

The study had a low participation rate (9.9%). This was probably due to a combination of factors. Employees with frequent SA may have a low intrinsic urgency to undertake action. The mild nature of most illnesses in frequent SA [50] in combination with an invitation related to sickness episodes in the past are possible reasons for a low intrinsic urgency to take action. In addition, employees are unaware of the high risk of future SA [11]. Moreover, there was no extrinsic stimulus: participation was completely voluntary. Most employees thought that the manager or colleagues did not find frequent SA a problem. A different recruitment procedure could increase study participation. A recent systematic review found that personal approaches lead to higher participation rates [51]. Recruitment can be increased through personal invitations by the researchers, invitation at the time of the third SA episode, when an employee has increased awareness possibly leading to increased motivation, or more involvement from the employer [52] or employee representatives. Due to scientific ethical codes (voluntary participation), involvement of the employer may be difficult to implement in a research setting. Several EHI studies found that recruitment using social media such as Facebook is more successful than more traditional recruitment strategies such as advertising [53,54]. However, such strategies seem difficult to apply to the population of employees with frequent SA. A combination of recruitment methods might also be more successful than relying solely on 1 method [55]. As it still may be difficult to reach participation rates as found in occupational health surveys (40% to 60%) and in earlier studies by Kant et al (50.2% questionnaire responders and 89% follow-up of [OP] intervention [15]) and Taimela et al (48% questionnaire responders and 68% attended the consultation at the OHS [14]), recruitment in intervention studies to reduce frequent SA should consider using a larger source population than the one used in this study. A higher participation rate combined with a larger source population may improve generalizability, decrease differences in characteristics of the participants between study arms, and increase power.

The adherence to the intervention tool was low. Reasons mentioned by participants were already having taken (a lot of) actions before the study or still undertaking actions, not seeing the added value of the intervention when knowing the problem not being work-related, being too busy, feeling low urgency, and being optimistic about their future health and SA. These reasons can be summarized as “low motivation to take (further) action.” To increase adherence, these issues have to be addressed. With little internal motivation, some external information or stimulus is needed. Studies on EHIs have found that human support increases adherence to the intervention [48,56] and effectiveness [49]. Increased adherence may be a more detailed explanation of the finding of Hutchesson in a meta-analysis [49] that direct human contact seems to intensify the effect of eHealth technologies. An OP can support various phases of the motivational process. An OP can increase readiness to act by increasing awareness of the high future risk of long-term SA. In addition, an OP can support an employee to make a realistic “plan of action” or refer to other professionals where necessary. An employer could also provide stimulation (they do in practice), but this is not ethically acceptable in scientific studies. Additional possibilities to increase adherence to the tool are the usage of reminders [57] or extra technological components such as more interactive exercises [58].

To increase adherence to an OP consultation, it might be more effective to offer only blended care, with the OP consultation as the main intervention and the EHI in the supporting role, as preparation for the consultation. Personal invitations to the OP could also increase adherence [51]. Preferably, the invitation is to the OP of the organization the employee works for; alternatively, a visit to another OP might be offered. The invitation should make clear that employees are also welcome in case of nonwork-related problems: the involvement of an occupational health service may be the reason why some participants in this study saw no reason for further action as they had private problems.

Future intervention studies on frequent absentees should deal with possible selection bias toward participants with more severe conditions by, for example, stratifying into groups with and without chronic disease or with and without long-term SA at baseline. Future intervention studies on SA frequency among employees with frequent SA should include larger sample sizes. Future research should also take into account the large reduction of SA frequency in the control group in the follow-up year. An intervention effect of 1 SA episode seems too high compared with the median SA frequency in the follow-up year. An intervention effect of 0.5 or even lower should be considered in future power calculations.

As the reasons for SA are very broad, it may be easier to develop an intervention focused on a specific disease than an intervention to reduce SA. However, it is important to continue to address reduction of SA in effectiveness studies. Not only is registered SA a very objective, numerical measurement that directly reflects economic costs (lost working days) but reduction of future frequent and long-term SA also focuses on prevention of any disease and ill-health.

### Conclusions

To our knowledge, this is the first RCT to examine the effect of an EHI tool on employees with frequent SA. When comparing the intervention groups with the control group, we found no significant effect of the intervention on SA frequency. Moreover, we found no significant effect on total SA days, burnout, engagement, or work ability. Only few employees with frequent SA participated in the study and relatively few took additional action to reduce frequent SA, with no apparent difference between the intervention groups and the control group. None of the participants set up a “plan of action” aimed at reducing SA frequency. Due to low adherence to the OP consultation, the results on the EHI-OP study arm do not represent results from blended care. Future research should test the effect of blended care, possibly involving the employer or manager of the employee with frequent SA to encourage adherence to an OP consultation. This could help to increase awareness and sense of urgency and may lead to more focus and adequate (local professional) and appropriate support.

## Appendix

Multimedia Appendix 1 Informed consent form.

Multimedia Appendix 2 CONSORT‐EHEALTH checklist (V 1.6.1).

Multimedia Appendix 3 Intervention elements.

Multimedia Appendix 4 Occupational physician instructions.

## Abbreviations

CAU: care as usual
eHealth: electronic health
EHI: eHealth intervention
EHI-only: study arm with only eHealth intervention
EHI-OP: study arm with eHealth intervention and invitation for occupational physician consultation
GP: general practitioner
IQR: interquartile range
METc: medical ethics committee
NVAB: Netherlands Society for Occupational Physicians
OP: occupational physician
OHS: occupational health service
RCT: randomized controlled trial
SA: sickness absence
UBOS: 9-item Utrecht Burnout Scale
UWES: 9-item Utrecht Work Engagement Scale
WAI: Work Ability Index

## References

[1] Virtanen M, Kivimäki M, Vahtera J, Elovainio M, Sund R, Virtanen P, Ferrie JE (2006). Sickness absence as a risk factor for job termination, unemployment, and disability pension among temporary and permanent employees. Occup Environ Med.

[2] Monitorarbeid.

[3] Koopmans PC, Roelen CA, Groothoff JW (2008). Risk of future sickness absence in frequent and long-term absentees. Occup Med (Lond).

[4] Eakin J, MacEachen E (2001). Health and the social relations of work: a study of the health-related experiences of employees in small workplaces. Sociol Health Ill.

[5] Koopmans PC, Roelen CA, Groothoff JW (2008). Frequent and long-term absence as a risk factor for work disability and job termination among employees in the private sector. Occup Environ Med.

[6] Beemsterboer W, Stewart R, Groothoff J, Nijhuis F (2009). A literature review on sick leave determinants (1984-2004). Int J Occup Med Environ Health.

[7] Roelen CA, Schreuder JA, Koopmans PC, Moen BE, Groothoff JW (2009). Sickness absence frequency among women working in hospital care. Occup Med (Lond).

[8] ten Brummelhuis LL, ter Hoeven CL, de Jong MD, Peper B (2012). Exploring the linkage between the home domain and absence from work: health, motivation, or both?. J Organiz Behav.

[9] Roskes K, Donders NC, van der Gulden JW (2005). Health-related and work-related aspects associated with sick leave: a comparison of chronically ill and non-chronically ill workers. Int Arch Occup Environ Health.

[10] Notenbomer A, Groothoff JW, van Rhenen RW, Roelen CA (2015). Associations of work ability with frequent and long-term sickness absence. Occup Med (Lond).

[11] Notenbomer A, Roelen CA, van Rhenen W, Groothoff JW (2016). Focus group study exploring factors related to frequent sickness absence. PLoS One.

[12] Arends I, van der Klink JJ, van Rhenen W, de Boer MR, Bültmann U (2014). Prevention of recurrent sickness absence in workers with common mental disorders: results of a cluster-randomised controlled trial. Occup Environ Med.

[13] Duijts SF, Kant I, van den Brandt PA, Swaen GM (2008). Effectiveness of a preventive coaching intervention for employees at risk for sickness absence due to psychosocial health complaints: results of a randomized controlled trial. J Occup Environ Med.

[14] Taimela S, Malmivaara A, Justén S, Läärä E, Sintonen H, Tiekso J, Aro T (2008). The effectiveness of two occupational health intervention programmes in reducing sickness absence among employees at risk. Two randomised controlled trials. Occup Environ Med.

[15] Kant I, Jansen NW, van Amelsvoort LG, van Leusden R, Berkouwer A (2008). Structured early consultation with the occupational physician reduces sickness absence among office workers at high risk for long-term sickness absence: a randomized controlled trial. J Occup Rehabil.

[16] Bolier L, Haverman M, Kramer J, Westerhof GJ, Riper H, Walburg JA, Boon B, Bohlmeijer E (2013). An Internet-based intervention to promote mental fitness for mildly depressed adults: randomized controlled trial. J Med Internet Res.

[17] Newhouse N, Martin A, Jawad S, Yu L, Davoudianfar M, Locock L, Ziebland S, Powell J (2016). Randomised feasibility study of a novel experience-based internet intervention to support self-management in chronic asthma. BMJ Open.

[18] van Genugten L, van Empelen P, Boon B, Borsboom G, Visscher T, Oenema A (2012). Results from an online computer-tailored weight management intervention for overweight adults: randomized controlled trial. J Med Internet Res.

[19] van Beugen S, Ferwerda M, Hoeve D, Rovers MM, Spillekom-van KS, van Middendorp H, Evers AW (2014). Internet-based cognitive behavioral therapy for patients with chronic somatic conditions: a meta-analytic review. J Med Internet Res.

[20] Beishuizen CR, Stephan BC, van Gool WA, Brayne C, Peters RJ, Andrieu S, Kivipelto M, Soininen H, Busschers WB, Moll VC, Richard E (2016). Web-based interventions targeting cardiovascular risk factors in middle-aged and older people: a systematic review and meta-analysis. J Med Internet Res.

[21] Riper H, Spek V, Boon B, Conijn B, Kramer J, Martin-Abello K, Smit F (2011). Effectiveness of E-self-help interventions for curbing adult problem drinking: a meta-analysis. J Med Internet Res.

[22] Beiwinkel T, Eißing T, Telle N, Siegmund-Schultze E, Rössler W (2017). Effectiveness of a web-based intervention in reducing depression and sickness absence: randomized controlled trial. J Med Internet Res.

[23] Ebert DD, Lehr D, Boß L, Riper H, Cuijpers P, Andersson G, Thiart H, Heber E, Berking M (2014). Efficacy of an internet-based problem-solving training for teachers: results of a randomized controlled trial. Scand J Work Environ Health.

[24] Ebert DD, Heber E, Berking M, Riper H, Cuijpers P, Funk B, Lehr D (2016). Self-guided internet-based and mobile-based stress management for employees: results of a randomised controlled trial. Occup Environ Med.

[25] Ebert DD, Lehr D, Heber E, Riper H, Cuijpers P, Berking M (2016). Internet- and mobile-based stress management for employees with adherence-focused guidance: efficacy and mechanism of change. Scand J Work Environ Health.

[26] Heber E, Lehr D, Ebert DD, Berking M, Riper H (2016). Web-based and mobile stress management intervention for employees: results of a randomised controlled trial. J Med Internet Res.

[27] Volker D, Zijlstra-Vlasveld MC, Anema JR, Beekman AT, Brouwers EP, Emons WH, van Lomwel AG, van der Feltz-Cornelis CM (2015). Effectiveness of a blended web-based intervention on return to work for sick-listed employees with common mental disorders: results of a cluster randomized controlled trial. J Med Internet Res.

[28] Demerouti E, Bakker AB, Nachreiner F, Schaufeli WB (2001). The job demands-resources model of burnout. J Appl Psychol.

[29] Clausen T, Burr H, Borg V (2014). Do psychosocial job demands and job resources predict long-term sickness absence? An analysis of register-based outcomes using pooled data on 39,408 individuals in four occupational groups. Int Arch Occup Environ Health.

[30] Slany C, Schütte S, Chastang J, Parent-Thirion A, Vermeylen G, Niedhammer I (2014). Psychosocial work factors and long sickness absence in Europe. Int J Occup Environ Health.

[31] Borritz M, Christensen KB, Bültmann U, Rugulies R, Lund T, Andersen I, Villadsen E, Diderichsen F, Kristensen TS (2010). Impact of burnout and psychosocial work characteristics on future long-term sickness absence. Prospective results of the Danish PUMA Study among human service workers. J Occup Environ Med.

[32] Schaufeli WB, Bakker AB, Van Rhenen W (2009). How changes in job demands and resources predict burnout, work engagement, and sickness absenteeism. J Organiz Behav.

[33] nvab-online.

[34] Eysenbach G, CONSORT-EHEALTH Group (2011). CONSORT-EHEALTH: improving and standardizing evaluation reports of Web-based and mobile health interventions. J Med Internet Res.

[35] Wet verbetering Poortwachter.

[36] van Dierendonck S (2000). Handleiding van de Utrechtse Burnout Schaal (UBOS) (Manual Utrecht Burnout Scale).

[37] Schaufeli WB (2006). The measurement of work engagement with a short questionnaire: a cross-national study. Educ Psychol Meas.

[38] Jääskeläinen A, Kausto J, Seitsamo J, Ojajärvi A, Nygård C, Arjas E, Leino-Arjas P (2016). Work ability index and perceived work ability as predictors of disability pension: a prospective study among Finnish municipal employees. Scand J Work Environ Health.

[39] El Fassi Mehdi, Bocquet V, Majery N, Lair ML, Couffignal S, Mairiaux P (2013). Work ability assessment in a worker population: comparison and determinants of Work Ability Index and Work Ability score. BMC Public Health.

[40] Van Veldhoven M, Meijman T (1994). Het meten van psychosociale arbeidsbelasting met een vragenlijst.

[41] Halford C, Wallman T, Welin L, Rosengren A, Bardel A, Johansson S, Eriksson H, Palmer E, Wilhelmsen L, Svärdsudd K (2012). Effects of self-rated health on sick leave, disability pension, hospital admissions and mortality. A population-based longitudinal study of nearly 15,000 observations among Swedish women and men. BMC Public Health.

[42] DeSalvo KB, Fan VS, McDonell MB, Fihn SD (2005). Predicting mortality and healthcare utilization with a single question. Health Serv Res.

[43] Bush K, Kivlahan DR, McDonell MB, Fihn SD, Bradley KA (1998). The AUDIT alcohol consumption questions (AUDIT-C): an effective brief screening test for problem drinking. Ambulatory Care Quality Improvement Project (ACQUIP). Alcohol Use Disorders Identification Test. Arch Intern Med.

[44] Kemper H, Ooijendijk W, Stiggelbout M (2000). Consensus over de nederlandse norm voor gezond bewegen.

[45] Douwes M, Hildebrandt V (2000). Vragen naar de mate van lichamelijke activiteit; onderzoek naar de test-hertest betrouwbaarheid en congruente validiteit van een vragenlijst.

[46] Faul F, Erdfelder E, Lang A, Buchner A (2007). G*Power 3: a flexible statistical power analysis program for the social, behavioral, and biomedical sciences. Behav Res Methods.

[47] Random Integer Generator.

[48] Heber E, Ebert DD, Lehr D, Cuijpers P, Berking M, Nobis S, Riper H (2017). The benefit of web- and computer-based interventions for stress: a systematic review and meta-analysis. J Med Internet Res.

[49] Hutchesson MJ, Rollo ME, Krukowski R, Ells L, Harvey J, Morgan PJ, Callister R, Plotnikoff R, Collins CE (2015). eHealth interventions for the prevention and treatment of overweight and obesity in adults: a systematic review with meta-analysis. Obes Rev.

[50] Hörnquist JO, Hansson B, Leijon M, Mikaelsson B (1990). Repeated short-term sick-leave and quality of life. An evaluation of a clinical socio-medical intervention. Scand J Soc Med.

[51] van Zon SK, Scholtens S, Reijneveld SA, Smidt N, Bültmann U (2016). Active recruitment and limited participant-load related to high participation in large population-based biobank studies. J Clin Epidemiol.

[52] Arends I, Bültmann U, Shaw WS, van Rhenen W, Roelen C, Nielsen K, van der Klink JJ (2014). How to engage occupational physicians in recruitment of research participants: a mixed-methods study of challenges and opportunities. J Occup Rehabil.

[53] Leonard A, Hutchesson M, Patterson A, Chalmers K, Collins C (2014). Recruitment and retention of young women into nutrition research studies: practical considerations. Trials.

[54] Watson NL, Mull KE, Heffner JL, McClure JB, Bricker JB (2018). Participant recruitment and retention in remote eHealth intervention trials: methods and lessons learned from a large randomized controlled trial of two web-based smoking interventions. J Med Internet Res.

[55] Hou S, Charlery SR, Roberson K (2014). Systematic literature review of internet interventions across health behaviors. Health Psychol Behav Med.

[56] Mohr DC, Cuijpers P, Lehman K (2011). Supportive accountability: a model for providing human support to enhance adherence to eHealth interventions. J Med Internet Res.

[57] Fry JP, Neff RA (2009). Periodic prompts and reminders in health promotion and health behavior interventions: systematic review. J Med Internet Res.

[58] Hasson H, Brown C, Hasson D (2010). Factors associated with high use of a workplace web-based stress management program in a randomized controlled intervention study. Health Educ Res.

