# Supplementary material for: Effect of an eHealth Intervention to Reduce Sickness Absence Frequency Among Employees With Frequent Sickness Absence: Randomized Controlled Trial
Source: J Med Internet Res. 2018 Oct 23;20(10):e10821. doi: 10.2196/10821 (PMC6231854; doi:10.2196/10821)
Supplement: Multimedia Appendix 1 [file jmir_v20i10e10821_app1.pdf]

Onderzoek naar effect van nieuw internet-hulpmiddel op gezondheid en verzuim

Geachte heer, mevrouw,

Arboned, de arbodienst van uw werkgever, werkt samen met het Universitair Medisch Centrum Groningen (UMCG) aan de ontwikkeling van een internet-hulpmiddel ten behoeve van de gezondheidsbevordering van medewerkers. Hiertoe zijn werkgevers benaderd om deel te nemen. De NHL heeft in het belang van de gezondheid van haar medewerkers medewerking toegezegd. Dit betekent dat alle medewerkers die zich in het afgelopen jaar 3 of meer keer zich hebben ziek gemeld benaderd worden met het onderstaande verzoek.

U heeft zich het afgelopen jaar drie of meer keer ziek gemeld bij uw werkgever. Een paar keer per jaar verzuimen lijkt niet zo erg, maar toch is frequent verzuim vaak een teken dat iemand niet in balans is.
Uit eerder onderzoek is gebleken dat mensen die 3 keer of vaker verzuimen een grotere kans hebben om langdurig te verzuimen.

De reden dat mensen verzuimen varieert: elke medische, privé en -werksituatie is weer anders. Uit gesprekken met werknemers die vaker verzuimen (via onderzoek met zogeheten focusgroepen) bleek dat mensen zich minder vaak ziek willen voelen en vaker gezond willen zijn. Ook willen ze zelf de regie op gezondheid en welzijn hebben.

Om die reden hebben wij een internet-hulpmiddel ontwikkeld dat u helpt te onderzoeken op welke manier ú gezondheidswinst kunt boeken. Wij willen graag weten hoe goed dit hulpmiddel is en wat uw mening erover is. Daarom vragen wij u om deel te nemen aan het onderzoek waarin we dit testen.
Hieronder leest u een aantal praktische zaken over wat dit onderzoek concreet voor u betekent, zoals de tijdsinvestering, de vertrouwelijkheid en voordelen van deelnemen aan het onderzoek.

Doel van het onderzoek
In dit onderzoek willen we weten hoe goed het internet-hulpmiddel is. We willen ook weten welke factoren van invloed zijn op het al dan niet effectief zijn van het hulpmiddel. Het uiteindelijke doel is om zoveel mogelijk mensen te helpen zich gezonder te voelen.

Procedure van het onderzoek
U heeft deze brief van uw werkgever ontvangen. Dit betekent dat uw werkgever graag mee wil werken aan dit onderzoek. U kunt zelf beslissen of u wel of niet mee wilt doen aan het onderzoek. Indien u mee wilt doen, kunt u het toestemmingsformulier in de bijgevoegde antwoord envelop terugsturen naar de onderzoeker.
U krijgt daarna een inlogcode toegestuurd samen met een kopie van het toestemmingsformulier.
Hiermee kunt u inloggen op een website, en meedoen aan het onderzoek. Er zijn 3 onderzoeksgroepen.
1 groep krijgt alleen de vragenlijst, 1 groep krijgt een advies op maat (digitaal) gebaseerd op de ingevulde vragenlijst. 1 groep krijgt advies op maat (digitaal) + een uitnodiging bij de bedrijfsarts. Uw indeling in een groep gebeurt via een soort loterij, wetenschappelijk genaamd ‘randomisatie’. Dit gebeurt al voordat u de vragenlijst invult. Kortom: u kunt niet beïnvloeden in welke groep u komt.

Indien u in de derde onderzoeksgroep zit, krijgt u een telefoonnummer via welke u een afspraak kunt maken bij de eigen bedrijfsarts voor een adviesgesprek. In dit gesprek wordt besproken hoe ú gezondheidswinst kunt krijgen door middel van het maken van een plan van aanpak. U kunt het gesprek zelf sturen met uw eigen vragen over werk of gezondheid. Maar het kan ook een gezamenlijk verkennend gesprek zijn over de factoren die een rol spelen bij uw gezondheid en welzijn en hoe u daar grip op kunt krijgen.

De deelnemers die het advies op maat hebben gekregen, krijgen na 3 maanden een zeer korte vragenlijst met de vraag wat u van het internet-hulpmiddel vond, hoe bruikbaar de adviezen voor u waren en welk effect het op uw leven en gezondheid heeft gehad. Diegenen die ook een uitnodiging hadden gekregen voor een adviesgesprek bij de bedrijfsarts, wordt ook gevraagd of ze geweest zijn en wat hun mening is over dit adviesgesprek.

Na 1 jaar krijgt u nog een korte vragenlijst: we willen dan graag weten hoe het met u gaat.
U krijgt dezelfde vragenlijst nog een keer toegezonden na 2 jaar.

Uw werkgever speelt geen verdere rol in dit onderzoek, behalve het overhandigen van deze uitnodigingsbrief voor deelname aan het onderzoek. Uw werkgever wordt ook niet op de hoogte gehouden. Zie verder ook hieronder bij vertrouwelijkheid van gegevens.

Voor en nadelen van het onderzoek voor u
Er zijn geen nadelen verbonden aan het onderzoek. De eerste vragenlijst invullen kost u de meeste tijd, ongeveer 20-30 minuten. De volgende vragenlijsten (na 3 maanden, 1 jaar en 2 jaar) kosten u hooguit 5-10 minuten om in te vullen.

Er is geen gezondheidsrisico voor u. Dit is de reden dat de METc (medisch ethische commissie van het UMCG) ontheffing heeft verleend van de wettelijk voorgeschreven verzekeringsplicht voor proefpersonen[1].

Voordelen voor u: u krijgt meer inzicht in hoe u uw gezondheid positief kunt beïnvloeden. U beslist uiteraard zelf wat u hier verder mee doet.

Vertrouwelijkheid van gegevens
De gegevens die in het kader van dit onderzoek worden verzameld worden strikt vertrouwelijk behandeld. De vragenlijsten worden apart verwerkt, waarbij alleen uw inlogcode bekend is en niet uw naam of andere persoonlijke gegevens. De gegevens worden dus gecodeerd verwerkt en zijn géén onderdeel van het medisch dossier. **De gegevens gaan dus niet naar uw werkgever, uw bedrijfsarts of anderen binnen de arbodienst.** De werkgever, bedrijfsarts en arbodienst weten niet wie wel en wie niet deelneemt aan het onderzoek. Zij zijn alleen op de hoogte dát er een onderzoek plaatsvindt. Als u in de onderzoeksgroep zit waarbij u bent ingedeeld voor een adviesgesprek met de bedrijfsarts, dan krijgt u een telefoonnummer om de afspraak zelf te maken. De bedrijfsarts raakt dus alleen op de hoogte van uw deelname aan het onderzoek als u zelf een afspraak maakt voor een gesprek. De bedrijfsarts heeft echter ook dan geen toegang tot de gegevens van het onderzoek. Het is aan u of u gaat en om te bepalen wat u in een dergelijk gesprek bespreekt.
Hierbij kunt u uiteraard wel de adviezen van het internet instrument meenemen.

Na 1 en 2 jaar vragen we bij de arbodienst ArboNed de verzuimcijfers op voor de periode van 1 respectievelijk 2 jaar. Na deze 2 jaar is het onderzoek afgerond. Andere gegevens, zoals bv de reden van verzuim of andere medische gegevens worden niet opgevraagd bij ArboNed
.
Vrijwilligheid van deelname
U bent er geheel vrij in al dan niet deel te nemen aan dit onderzoek. Verder heeft u altijd, ook al bent u wel begonnen met het onderzoek, het recht om zonder opgaaf van redenen af te zien van verdere deelname van het onderzoek. Deze beslissing zal geen nadelige gevolgen hebben op uw (mogelijk toekomstige) begeleiding door de arbodienst of vanuit uw werkgever. Uw werkgever en bedrijfsarts worden niet op de hoogte gesteld als u niet deelneemt of indien u niet meer wilt deelnemen in de toekomst.

Deelname aan het onderzoek
U heeft deze informatiebrief van uw werkgever gekregen. Wij hopen dat u meedoet aan het onderzoek.
U kunt alleen deelnemen aan het onderzoek door ons het toestemmingsformulier ingevuld terug te sturen. Door ondertekening van het toestemmingsformulier stemt u in met deelname aan het onderzoek. Wij vragen u om ons binnen 1 maand het toestemmingsformulier toe te sturen. U krijgt vervolgens een inlogcode toegestuurd, waarmee u uw eigen deelname aan het onderzoek zelf kunt starten. Op het toestemmingsformulier staat ook een verzoek om uw e-mail adres. Wij benaderen u na 3 maanden, 1 jaar en 2 jaar via de e-mail voor het invullen van de korte vragenlijsten. Ook krijgen wij graag uw adresgegevens, zodat we u deze korte vragenlijsten ook kunnen toesturen indien u binnen 2 jaar verandert van e-mail adres.

Als u van werkgever verandert, dan moeten wij uw deelname aan het onderzoek helaas stoppen. U kunt ons dit tussentijds aangeven, of aangeven op het moment dat u gevraagd wordt om een volgend vragenformulier in te vullen. Indien u in een situatie van langdurig verzuim terechtkomt kunt u gewoon blijven deelnemen.

Nadere informatie
Mocht u na het lezen van deze brief nog nadere informatie willen, dan kunt u altijd contact opnemen met de bedrijfsarts-onderzoeker A. Notenbomer. U kunt haar bereiken via e-mail: a.notenbomer@umcg.nl of 058 - 233 92 33. Ook kunt u contact opnemen met de onafhankelijke arts, J. Buitenhuis, die niet bij het onderzoek betrokken is, maar wel op de hoogte. U kunt J. Buitenhuis bereiken op 06 – 536 791 89 of via J.Buitenhuis@unive.nl.

---

1 Wel heeft het UMCG zelf een algemene aansprakelijkheidsverzekering, geldig voor deelnemers aan onderzoeken

TOESTEMMINGSVERKLARING

Voor deelname aan het wetenschappelijk onderzoek: een onderzoek naar een nieuw internet hulpmiddel voor gezondheid en verzuim

Wij vragen u onderstaande verklaring in te vullen, te ondertekenen en terug te sturen in bijgevoegde envelop. Een postzegel is niet nodig.

- Ik ben naar tevredenheid over het onderzoek geïnformeerd. Ik heb de schriftelijke informatie goed gelezen. Ik ben in de gelegenheid gesteld om vragen over het onderzoek te stellen. Mijn vragen zijn naar tevredenheid beantwoord. Ik heb goed over deelname aan het onderzoek kunnen nadenken. Ik heb het recht mijn toestemming op ieder moment weer in te trekken zonder dat ik daarvoor een reden behoef op te geven.

- Ik stem toe met deelname aan het onderzoek.

Naam :

Voorletter(s) :

Geslacht : man 0 vrouw 0 (aankruisen wat van toepassing is)

Geboortedatum :

Adres :

Postcode :

Woonplaats :

E-mail adres :

Handtekening : Datum:

- Ondergetekende verklaart dat de hierboven genoemde persoon schriftelijk over het bovenvermelde onderzoek geïnformeerd is. Hij/zij verklaart tevens dat een voortijdige beëindiging van de deelname door bovengenoemde persoon van geen enkele invloed zal zijn op de zorg die hem of haar toekomt.

Naam : A. Notenbomer
Functie : Onderzoeker UMCG/RuG, Bedrijfsarts ArboNed
Handtekening :

Datum: 5-12-13

rijksuniversiteit groningen ArboNed umcg
