# Supplementary material for: Effect of an eHealth Intervention to Reduce Sickness Absence Frequency Among Employees With Frequent Sickness Absence: Randomized Controlled Trial
Source: J Med Internet Res. 2018 Oct 23;20(10):e10821. doi: 10.2196/10821 (PMC6231854; doi:10.2196/10821)
Supplement: Multimedia Appendix 3 [file jmir_v20i10e10821_app3.pdf]

Items	Intervention elements
Job demands[a]	General advice for self-management
	Referral to communicate with the manager, colleagues, human resource management (HRM), and possibly the works council, the OP or social worker when someone needs support
	Link to documents (depending on item): ‘grip on problems’, ‘tips for difficult conversations’, ‘useful addresses’, ‘out of balance, in balance’. The latter included links to several other documents.
Job resources[b]	General advice for self-management
	Referral to communicate with the manager, colleagues, HRM, and possibly the OP or social worker when someone needs support
Burn out and engagement	General advice for self-management
	Referral to OP, psychologist, social work, manager, HRM through documents
	Link to documents ‘out of balance, in balance’, ‘grip on problems’, that also refer to some of the other documents
Work ability	General advice for self-management (taking into account whether low work ability is or is not related to a chronic disease)
	Referral to manager (work changes), GP, specialist, rehabilitation specialist, OP, paramedics (focus on increase of work ability through therapy or treatment)
General health and chronic disease	General advice for self-management
	Referral to manager, colleagues (work related), GP, OP , social worker
	Link to documents: ‘out of balance, in balance,’, ‘useful addresses’, ‘rehabilitation principles’, health self-management tips’’ .
Psychological health	General advice for self-management
	Referral to websites (www.trimbos.nl), www.kleurjeleven.nl, www.mentaalvitaal.nl), and practitioners (psychologist, social worker, confidential mediator, GP, OP), manager or HRM (work related)
	Link to documents: ‘out of balance, in balance’, ‘grip on problems’, ‘decrease worrying’ and through-links to other documents.
Lifestyle and BMI	General advice for self-management
	Referral to informative websites (e.g. www.alcoholinfo.nl),
	Link to document ‘useful addresses’ that include amongst many others GP, OP, paramedics, websites on financial issues, informal care) etc.

a : work pace, emotional demands and work-home interference, physical demands

b: feedback, learning opportunities, supervisor support, co-worker support and autonomy
