# Supplementary material for: Effect of an eHealth Intervention to Reduce Sickness Absence Frequency Among Employees With Frequent Sickness Absence: Randomized Controlled Trial
Source: J Med Internet Res. 2018 Oct 23;20(10):e10821. doi: 10.2196/10821 (PMC6231854; doi:10.2196/10821)
Supplement: Multimedia Appendix 4 [file jmir_v20i10e10821_app4.pdf]

Informatie BA ten behoeve van adviesgesprek bij de bedrijfsarts

Kader: Interventieonderzoek onder frequent verzuimers.

- Doel van de studie: effectiviteit testen van de tool en ook verschil in effectiviteit tussen alleen de internet-tool of internet-tool + afspraak bij BA.

- Internet tool = zelf management tool.

- Doel voor mensen: hoe kan ik gezondheidswinst verkrijgen?
  En dus niet: hoe kan ik minder verzuimen! Dit n.a.v. de focusgroepen: mensen vinden minder verzuimen alleen interessant als dit het gevolg is van minder vaak zich ziek voelen. Daarnaast: ze wilden zelf grip hebben.

- 4 redenen voor een werknemer om in het kader van het onderzoek de bedrijfsarts te bezoeken:

  - Groep 1: controle groep: heeft alleen een vragenlijst gekregen. Geen advies gekregen. Is wakker geschud, gaat toch praten.
  - Groep 2: interventie groep: internet-tool: zij kunnen een individueel advies hebben gekregen om met de BA te overleggen. Vaak is dit een advies in 2e aanleg, na eerst advies om met lg of P&O te gaan praten.
  - Groep 3: interventie groep: internet-tool + BA adviesgesprek. Deze groep heeft dus aparte mail gekregen dat ze in deze interventie groep zitten en krijgt een telefoonnummer van het secretariaat/RPA's + naam eigen bedrijfsarts.
  - Reden 4: leidinggevende heeft iemand naar aanleiding van het onderzoek toch gepusht om naar BA te gaan.

- Boeken: 121: open spreekuur. Gratis! Iemand die buiten het onderzoek om komt, of gestuurd wordt door de werkgever, dat is de gewone code.

- Geheimhouding is zeer belangrijk!: de werkgever mag niet op de hoogte gebracht worden DAT werknemer is geweest. Ook mag de

werkgever geen informatie krijgen. TENZIJ dit expliciet met werknemer is besproken en dit ook echt in het belang van de werknemer is. Dus geen brieven of tel. gesprekken met werkgever met bv: ik zie geen medische reden voor frequent verzuim van deze werknemer. In feite moet je het als een huisarts spreekuur bekijken: wat heeft werknemer nodig om gezonder te worden (en uiteindelijk minder vaak te verzuimen). Daarin ondersteunen.

- Doel van het adviesgesprek bij BA (deze informatie heeft werknemer ook gekregen):
  - Werknemer heeft vragen over gezondheid en werk
  - Verkennend gesprek over hoe werknemer invloed op zijn gezondheid kan krijgen. Een Plan van Aanpak kan dan (eventueel) gemaakt worden.

- Actie bedrijfsarts na adviesgesprek: Graag Noteren wie is geweest en waar deze persoon werkt. Zie bijgevoegd formulier. naam persoon. Was werknemer zelf actief in de communicatie in het spreekuur? (kortom: had werknemer vragen? Of was het meer een verplicht nummer?). Tevens: was werknemer al aan de slag voorafgaand aan het spreekuur om iets te doen waardoor hij/zij verwacht gezonder te worden? Of, verwacht je dat werknemer na het spreekuur iets gaat doen?
